# Minimal Number of Required Inputs for Temporally Precise Action Potential Generation in Auditory Brainstem Nuclei

**DOI:** 10.3389/fncel.2020.592213

**Published:** 2020-11-05

**Authors:** Nikolaos Kladisios, Linda Fischer, Felix Felmy

**Affiliations:** Institute of Zoology, University of Veterinary Medicine, Hannover, Germany

**Keywords:** auditory brainstem, postnatal development, action potential generation, synaptic transmission, superior olive, lateral lemniscus

## Abstract

The auditory system relies on temporal precise information transfer, requiring an interplay of synchronously activated inputs and rapid postsynaptic integration. During late postnatal development synaptic, biophysical, and morphological features change to enable mature auditory neurons to perform their appropriate function. How the number of minimal required input fibers and the relevant EPSC time course integrated for action potential generation changes during late postnatal development is unclear. To answer these questions, we used *in vitro* electrophysiology in auditory brainstem structures from pre-hearing onset and mature Mongolian gerbils of either sex. Synaptic and biophysical parameters changed distinctively during development in the medial nucleus of the trapezoid body (MNTB), the medial superior olive (MSO), and the ventral and dorsal nucleus of the lateral lemniscus (VNLL and DNLL). Despite a reduction in input resistance in most cell types, all required fewer inputs in the mature stage to drive action potentials. Moreover, the EPSC decay time constant is a good predictor of the EPSC time used for action potential generation in all nuclei but the VNLL. Only in MSO neurons, the full EPSC time course is integrated by the neuron’s resistive element, while otherwise, the relevant EPSC time matches only 5–10% of the membrane time constant, indicating membrane charging as a dominant role for output generation. We conclude, that distinct developmental programs lead to a general increase in temporal precision and integration accuracy matched to the information relaying properties of the investigated nuclei.

## Introduction

Neurons in the auditory brainstem form specialized structure-function relationships. To accommodate their individual functions, neurons refine their biophysical and synaptic properties during postnatal development in a distinct manner. Thus, mature neurons in the auditory nuclei perform specific processing tasks based on their connectivity, and biophysical and synaptic features. Neurons of the medial superior olive (MSO) for example act as ultra-fast coincidence detectors integrating binaural time differences (Goldberg and Brown, 1969; Yin and Chan, 1990). Neurons of the dorsal nucleus of the lateral lemniscus (DNLL) integrate over longer time windows to generate a long-lasting suppression acting as a temporal binaural filter (Yang and Pollak, 1994; Pecka et al., 2007; Meffin and Grothe, 2009; Siveke et al., 2019). The large glutamatergic somatic synapses in the medial nucleus of the trapezoid body (MNTB; Held, 1893; Forsythe, 1994) and the ventral nucleus of the lateral lemniscus (VNLL; Stotler, 1953; Adams, 1997; Berger et al., 2014) allow for rapid and temporally precise feed-forward inhibition.

During late postnatal development distinct cell features change in auditory brainstem neurons. Often observed is a developmental drop in input resistance (Magnusson et al., 2005; Scott et al., 2005; Chirila et al., 2007; Hoffpauir et al., 2010; Rusu and Borst, 2011; Franzen et al., 2015, 2020) based on the upregulation of ion channels (Scott et al., 2005; Hassfurth et al., 2009; Khurana et al., 2012; Franzen et al., 2015). In this developmental period synaptic current accelerates by changes in AMPA receptors (Bellingham et al., 1998; Taschenberger and von Gersdorff, 2000; Joshi and Wang, 2002; Koike-Tani et al., 2005; Youssoufian et al., 2005; Ammer et al., 2012; Felix and Magnusson, 2016; Baumann and Koch, 2017; Franzen et al., 2020) and a reduction or loss of synaptic NMDA receptors (Bellingham et al., 1998; Steinert et al., 2010; Case et al., 2011; Hirtz et al., 2011; Ammer et al., 2012; Winters and Golding, 2018; Franzen et al., 2020). The neuronal morphology remodels by changes in the dendritic arbor (Sanes et al., 1992; Rietzel and Friauf, 1998; Rautenberg et al., 2009) and somatic size (Franzen et al., 2015). Moreover, the axonal structure refines leading to fewer but often larger synaptic contact sites (Kim and Kandler, 2003; Werthat et al., 2008; Franzen et al., 2020). It is assumed that these developmental processes orchestrate the increase in temporal precision of neuronal input-output functions, a key feature in the auditory brainstem.

Neuronal input-output functions depend on the synaptically mediated input and the postsynaptic integration and action potential generation processes. Thereby, the relation between input size and integrational properties is crucial. For temporally precise supra-threshold excitation, the number of simultaneously activated input fibers sets the minimally required input conductance. This minimally required input conductance is modified by passive and active biophysical properties of the postsynaptic neurons, such as cell morphology, resting potential, input resistance, and voltage-gated ion channels. Crucial for synaptic integration is also the EPSC time course (Cathala et al., 2003; Axmacher and Miles, 2004; Rodriguez-Molina et al., 2007; Ammer et al., 2012; Franzen et al., 2015) and its relation to the membrane time constant. Fast EPSCs in neurons with slow membrane time constants may be restricted to drive the charging of the neuron, while slow EPSCs in neurons with fast membrane time constants will be largely integrated based on the resistive membrane element.

Since most of the crucial parameters for synaptically evoked input-output functions change during development, it is expected that these functions are not static during maturation. Moreover, as a comparative approach over several auditory nuclei is missing, common denominators or structure-specific features during development are difficult to identify. To identify common motifs and individual differences during late postnatal development in auditory brainstem nuclei, we quantified the synaptically evoked input-output functions in the VNLL, MNTB, MSO, and DNLL in prehearing [postnatal day (P) 9/10] and mature gerbils. Using strength-duration relationships of current injections together with afferent minimal fiber stimulation, we estimated the number of input fibers required for temporal precise action potential generation at rest and during ongoing activity. Dynamic clamp recordings were used to corroborate these estimates and identified postsynaptic contributions to action potential generation. Our data allowed us to determine the relevant time of the EPSC that is required during postsynaptic integration for the generation of an action potential. This comparative approach shows that changes during late postnatal developmental are distinct in different nuclei, generally rendering neurons more excitable so that fewer input fibers are required to generate postsynaptic action potentials. These developmental changes match the functional needs assigned to the neurons of each auditory structure.

## Materials and Methods

Mongolian gerbils (*Meriones unguiculatus*), based on Charles River background, were bred in the institute’s animal facility, kept in a 12 h light/dark cycle, and fed *ad libitum*. For this study, animals of either sex and the specified ages were used. All experiments that used animals were reviewed and approved by the university (licensed under TiHo-T-2019-4) and local authorities and were in accord with the German animal welfare law.

### Slice Preparation

Animals were anesthetized with isoflurane, then decapitated and the brains were rapidly removed in preparation solution consisting of (mM): 120 sucrose, 25 NaCl, 25 NaHCO_3_, 2.5 KCl, 1.25 NaH_2_PO_4_, 3 MgCl_2_, 0.1 CaCl_2_, 25 glucose, 0.4 ascorbic acid, 3 myo-inositol, and 2 Na-pyruvate, bubbled with 95% O_2_ and 5% CO_2_ with a pH of 7.4. Animals of P9 and 10 were defined as young animals and were prepared in ice-cold solutions. Mature animals (VNLL: P18–31, mean P22.8 ± 0.6; MNTB: P48–91, mean P61.5 ± 3; MSO: P49–104, mean P72.6 ± 3.4; DNLL: P28–72, mean P52.8 ± 2.4) were prepared in cold or warm solutions. After trimming the brainstem, 200 μm thick transversal slices (or 120 μm thick horizontal slices for adult MSO) were taken with a VT1200S vibratome (Leica), incubated for 45 min at 34°C and kept thereafter at room temperature in a recording solution containing (mM): 125 NaCl, 25 NaHCO_3_, 2.5 KCl, 1.25 NaH_2_PO_4_, 1 MgCl_2_, 1.2 or 2 CaCl_2_, 25 glucose, 0.4 ascorbic acid, 3 myo-inositol, and 2 Na-pyruvate, bubbled with 95% O_2_ and 5% CO_2_ with a pH of 7.4.

### Electrophysiology and Stimulation

Whole-cell recordings were performed at 34–36°C. Brainstem neurons were visualized with a Retiga 2000DC camera connected to a TILL Photonics system (FEI) and a monochromator (Polychrome V). Recordings were made with an EPC10/2 USB amplifier controlled by Patchmaster (HEKA). All potentials reported were corrected for liquid junction potential (LJP) according to Barry (1994) using a custom-written IGOR Pro script based on ionic concentration gradients between intra- and extra-cellular solutions. The internal solution for voltage-clamp contained (mM): 130 Cs-gluconate, 20 TEA-Cl, 10 HEPES, 5 Cs-EGTA, 5 Qx-314, 5 Na_2_-phosphocreatine, 4 Mg-ATP, 0.3 Na_2_-GTP and 0.1 spermine with a LJP of 13.3 mV. The internal solution for current-clamp contained (mM): 145 K-gluconate, 15 HEPES, 4.5 KCl, 7 Na_2_-phosphocreatine, 2 Mg-ATP, 0.5 K-EGTA, 2 K_2_-ATP, and 0.3 Na_2_-GTP with an LJP of 15.95 mV. The acquisition frequency was set at 50 Hz and data were filtered at 3 kHz. Electrode resistance was 3–5 MΩ and access resistance was compensated to a residual of 2 MΩ in voltage-clamp mode. In current and dynamic clamp the access resistance was bridge balanced to 100% and continuously monitored. No holding current was applied.

The firing behavior of neurons was evaluated by injecting hyper- and depolarizing currents (1 nA to −400 pA, 100 pA interval; 6 nA to −3 nA, 0.5 nA interval for adult MSO). Membrane properties of all neurons were extracted by injecting small sub-threshold, depolarizing square currents (5–10 pA; 50 pA for mature MSO), and averaging at least 50 repetitions. Membrane decay time (*t*_mem_) was calculated by fitting an exponential on the average traces at stimulus onset. Input resistance (*R*_in_) was estimated using Ohms law (*R*_in_ = *V*_ss_ − *E*_rest_/*I*), where *E*_rest_ is the resting potential and *V*_ss_ the voltage steady-state response. Strength-duration relationships were calculated by injecting square pulses of varying current (incremented in 30–100 pA steps) and duration (0.1–150 ms) until supra-threshold events were evoked for each stimulation duration. To estimate the minimally required stimulus strength we plotted the action potential evoking charge against the pulse duration and fitted a linear regression line. The slope equals the rheobase and the *x*-intercept divided by rheobase the chronaxie values according to the Weiss formula (Mogyoros et al., 1996). The voltage threshold was manually estimated at the current stimulus with the longest duration approximating the rheobase.

To minimally stimulate afferent fibers, a glass pipette filled with a recording solution was placed near the neuron and 200 μs long biphasic voltage pulses delivered by a Model 2100 A-M system stimulator and triggered by the EPS10/2 amplifier were applied. The stimulation voltage was adjusted to elicit EPSCs just above threshold, where failures also occurred. EPSCs were isolated by blocking inhibition with 1 μM strychnine and 10 μM SR95537. After detecting synaptic inputs, single fiber EPSC size and kinetics in response to step potentials ranging from −93 to 67 mV with 10 mV increments were recorded. Double exponential functions were fitted at the EPSC peak to calculate fast and weighted decay time {*t*_w_ = [*t*_1_ * amplitude (*t*_1_) + *t*_2_ * amplitude (*t*_2_)]/amplitude}. The rapid, maximum deflection was considered as the fast α-amino-3-hydroxy-5-methyl-4-isoxazolepropionic acid (AMPA) receptor (AMPAR) EPSC peak and residual current after 5 ms as the slow *N*-methyl-D-aspartic acid receptor (NMDAR) mediated current. Linear regression was fitted to the first eight negative step potentials (−93 to −23 mV). The rectification index (RI) was determined as the ratio of the measured AMPAR EPSC peak at 47 mV to the projected value of the linear fit at the same potential (Scheuss and Bonhoeffer, 2014). Non-linear kinetics of NMDAR were calculated by fitting Boltzmann sigmoid functions to NMDAR IV curves. Single fiber stimulation trains of 20 pulses at 10, 50, and 100 Hz for P9/10, and additionally 300 Hz for adult neurons were performed. For quantifying short-term plasticity (STP), we used the envelope current, i.e., the maximum EPSC deflection from baseline, to normalize pulse currents to the first peak. Steady-state depression of each neuron was measured as the average short-term depression (STD) at the last three stimuli.

For the dynamic clamp recordings, we applied the interface introduced by Yang et al. (2015), which allows the real-time integration of linear and non-linear synaptic conductances. To produce dynamic clamp templates, synaptically stimulated AMPAR and NMDAR mediated EPSC train responses from the same cells were isolated for each nucleus and age group. First, single afferent fibers were stimulated at −93 mV, where NMDARs are predominantly closed. Then, AMPARs were blocked with 20 μM DNQX, and NMDAR currents were recorded at 37 mV. Both AMPAR and NMDAR mediated responses to train stimulations were converted to conductance templates. During recordings, current components required to accommodate time-varying synaptic conductances of both receptors were calculated online (AMPAR: *I*_AMPA_ = *G*(*t*) (*V*_m_ − *V*_rev_); NMDAR: *I*_NMDA_ = (*V*_m_ − *V*_rev_) *G*_NMDA_(*t*)/(1 + exp[(*V*_half_ − *V*_m_)/*V*_rate_])) and injected in the neurons *via* different channels. With custom-written IGOR commands template amplitudes were increased to determine the required conductance for single supra-threshold events at the onset and the ongoing component of the stimulus.

While recording membrane properties in current clamp, and single pulse EPSC in voltage-clamp the recording solution contained 2 mM CaCl_2_. In contrast, 1.2 mM CaCl_2_ was used for train stimulations and dynamic clamp recordings to achieve more physiologically relevant conditions (Lorteije et al., 2009; Borst, 2010).

### Data Analysis and Statistics

Electrophysiological data were analyzed with IGOR Pro 7 (Wavemetrics) with custom-written procedures and further processed with Microsoft Excel. Statistical analysis was conducted with GraphPad Prism 8. As most data were non-normally distributed, they are presented as median, and first and third quartiles. Only STP data of Figure 5, and AMPAR and NMDAR mediated IV curves of Figure 6 are shown as mean ± standard error of means (SEM). Statistical comparison between age groups was performed with Mann–Whitney *U* and Student’s *t*-test, and between nuclei with Kruskal–Wallis and Welch’s ANOVA. The Wilcoxon signed-rank test was used for paired analysis between AMPAR and NMDAR conductance paradigms of Figure 7. The significance level was set at 0.05. For the results of current-clamp recordings, presented on Figures 2, 3, 39 animals were sacrificed (VNLL: P9/10; *N* = 4, mature; *N* = 3, MNTB: P9/10; *N* = 5, mature; *N* = 5, MSO: P9/10; *N* = 5, mature; *N* = 6, DNLL: P9/10; *N* = 7, mature; *N* = 4). For single-fiber EPSC recordings presented in Figures 4, 6, and train stimulation in Figure 5, a total of 37 (VNLL: P9/10; *N* = 3, mature; *N* = 6, MNTB: P9/10; *N* = 6, mature; *N* = 5, MSO: P9/10; *N* = 4, mature; *N* = 4, DNLL: P9/10; *N* = 4, mature; *N* = 5) and 49 animals (VNLL: P9/10; *N* = 5, mature; *N* = 15, MNTB: P9/10; *N* = 5, mature; *N* = 4, MSO: P9/10; *N* = 4, mature; *N* = 5, DNLL: P9/10; *N* = 6, mature; *N* = 5) were used, respectively. For the dynamic clamp results of Figures 7, 8, 34 gerbils (VNLL: P9/10; *N* = 3, mature; *N* = 3, MNTB: P9/10; *N* = 5, mature; *N* = 4, MSO: P9/10; *N* = 4, mature; *N* = 3, DNLL: P9/10; *N* = 5, mature; *N* = 7) were sacrificed.

**Figure 1:**
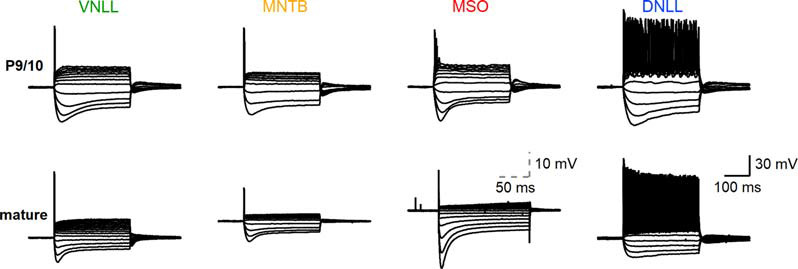
Biophysical input-output behavior of auditory brainstem neurons. Top: voltage-current relationships of P9/10 ventral nucleus of the lateral lemniscus (VNLL), medial nucleus of the trapezoid body (MNTB), medial superior olive (MSO), and dorsal nucleus of the lateral lemniscus (DNLL) neurons. Bottom: Same as the top, with mature neurons (VNLL: P27, MNTB: P54, MSO: P80, DNLL: P56). The scale of MSO neuron response was adjusted to visualize the small action potentials. Scale: 30 mV, 100 ms; scale MSO: 10 mV, 50 ms.

**Figure 2:**
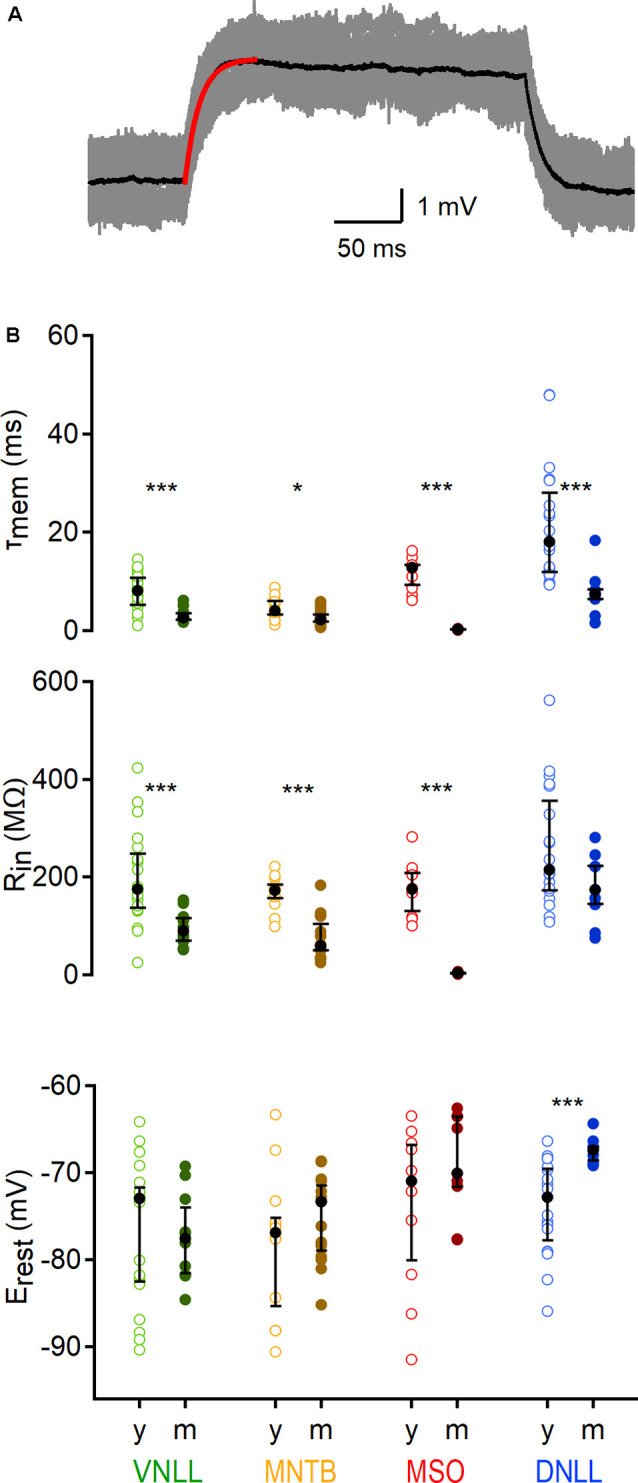
Developmental regulation of sub-threshold membrane properties of auditory brainstem neurons. **(A)** Average voltage deflection in response to small, depolarizing currents fitted with an exponential (red curve) to calculate sub-threshold properties. Scale: 1 mV, 50 ms. **(B)** Sub-threshold membrane properties of VNLL, MNTB, MSO, and DNLL neurons, between P9/10 (y) and >P18 (m). Top: Membrane time constant (*τ*_mem_), middle: input resistance (*R*_in_), bottom: resting potentials (*E*_rest_). Open symbols represent individual P9/10, closed symbols mature neurons. Nuclei are color-coded (VNLL: green, MNTB: orange, MSO: red, DNLL: blue; P9/10 as light and mature as dark-hued variations). y = data from young animals, m = data from mature animals. Number of recorded neurons for VNLL: y; *n* = 19, m; *n* = 10, MNTB: y; *n* = 12, m; *n* = 15, MSO: y; *n* = 10, m; *n* = 9, DNLL: y; *n* = 19, m; *n* = 9. Black symbols show the median with the first and third quartiles. *p < 0.05, ***p < 0.001.

**Figure 3:**
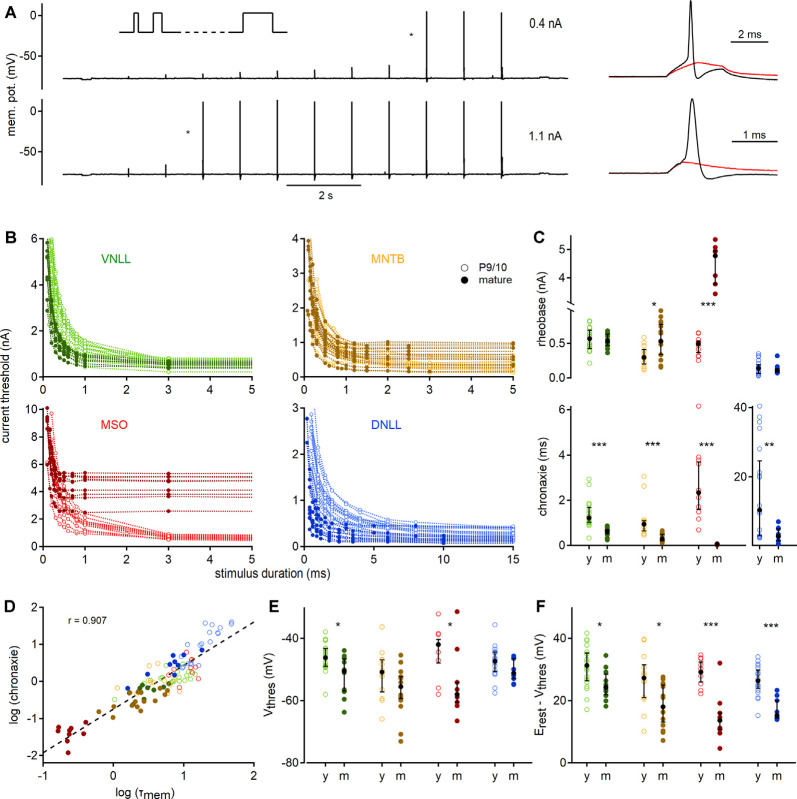
Quantification of action potential thresholds and neuronal excitability by strength-duration curves. **(A)** Stimulation paradigm; square currents of ascending duration (inset) were injected exemplified by a P27 VNLL neuron. In every stimulation round, the current amplitude was increased until action potentials were induced (right). Cell responses to current injections of 0.4 nA (top) and 1.1 nA (bottom) are shown. Traces on the right show zoomed stimuli from the protocols, denoted with asterisks. Red curves show the last sub-threshold, and black lines the first supra-threshold response. **(B)** Strength-duration curves for four nuclei in two age groups. Supra-threshold responses as a function of current intensity vs. stimulus duration. Data from P9/10 animals are shown as open symbols with a light color, from mature neurons as filled symbols. **(C)** Rheobase (top) and chronaxie values (bottom) extracted from strength-duration curves. **(D)** Correlation between chronaxie and membrane time constant (*τ*_mem_, *r* = 0.907). The regression line is shown as a dotted line. **(E)** Voltage threshold (*V*_thres_). **(F)** Potential (between *V*_thres_ and *V*_rest_) that needs to be crossed to reach the voltage threshold. Cell numbers per nucleus and age group, markers, and color-coding as in Figure 2. Black symbols median with first and third quartiles. *p < 0.05, **p < 0.01, ***p < 0.001.

**Figure 4:**
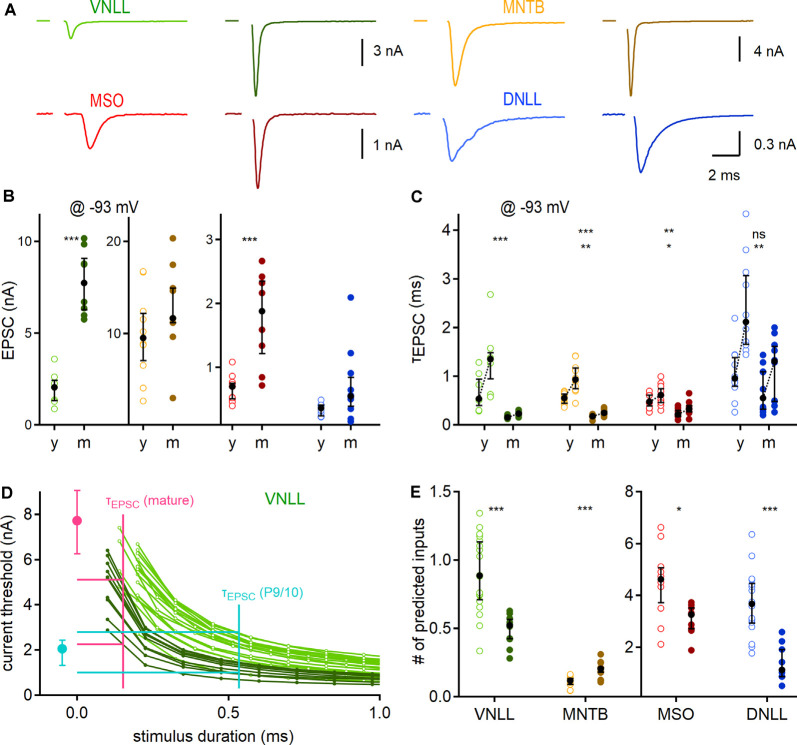
Developmental maturation of synaptic properties and predicted influence on output generation. **(A)** Exemplary EPSC forms of P9/10 (left) and mature animals (right; VNLL: P23, MNTB: P91, MSO: P65, DNLL: P63) of the four auditory brainstem nuclei. **(B)** Peak EPSC size, recorded at −93 mV. **(C)** Maturation of EPSC kinetics. Pair-wise fast (left) and weighted (right) decay time constants recorded at −93 mV. Asterisks on the top show the significance level of fast, and asterisks on the bottom of the weighted time constant values between P9/10 and mature neurons. **(D)** Strength-duration curves as a dose-response curve for inferring relevant threshold currents. The median fast decay time constant was used as a proxy for stimulus duration (x-axis) to infer the current threshold (y-axis) for action potential generation in strength-duration curves (vertical lines of cyan: P9/10; and magenta: mature). Median EPSC sizes for P9/10 (cyan) and mature VNLL neurons (magenta). Horizontal cyan and magenta lines show maximal and minimal inferred current thresholds. The strength-duration curves are reproduced as a magnification from Figure 3B. **(E)** Predicted minimum numbers of afferent fibers for postsynaptic supra-threshold excitation. Number of recorded neurons for VNLL: y; *n* = 7, m; *n* = 8, MNTB: y; *n* = 10, m; *n* = 9, MSO: y; *n* = 11, m; *n* = 8, DNLL: y; *n* = 10, m; *n* = 10. Markers and color-coding as in Figure 2. Black symbols show the median with the first and third quartiles. ns = not significant, *p < 0.05, **p < 0.01, ***p < 0.001.

**Figure 5:**
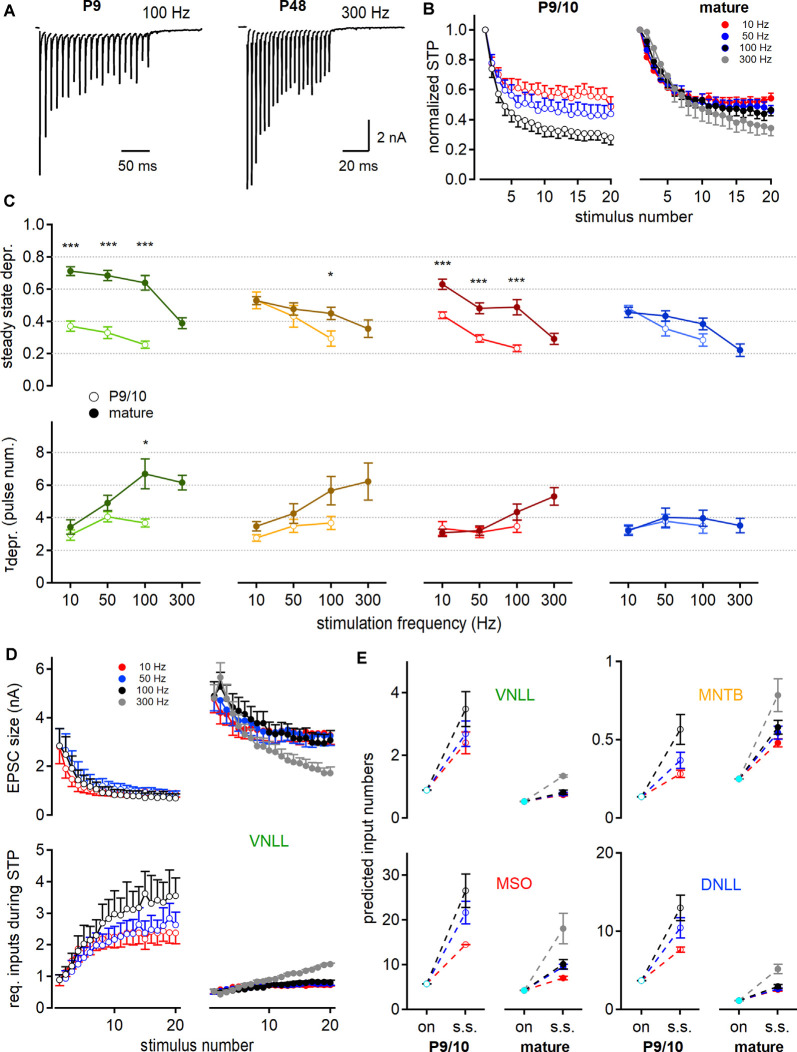
Short-term plasticity (STP) increases the predicted number of minimally required inputs for postsynaptic action potential generation. **(A)** EPSC responses to train stimulations evoked in young (left, P9) and mature (right, P48) MNTB neurons at 100 and 300 Hz, respectively. **(B)** Age- and frequency-dependence of STP profile in MNTB neurons. The amplitude of each pulse is estimated as a maximal deflection from baseline and normalized to the first response size. **(C)** Steady-state depression (top) and decay of depression (bottom) as a function of stimulation frequency. Steady-state depression is measured as the average STP of the last three stimulation pulses. **(D)** STP profile of EPSC size in P9/10 (left) and P23 (right) VNLL neurons during train stimulation (top). Bottom: Inferred minimal required input number to achieve action potential generation throughout the stimulation train. Predictions based on current amplitudes and initial estimates of input numbers from Figure 4. Standard error of means (SEM) displayed only one-sided for clarity. **(E)** Predicted input numbers of P9/10 (left) and mature (right) neurons at onset (cyan symbols) and during the steady-state depression. Number of recorded neurons for VNLL: y; *n* = 9, m; *n* = 16, MNTB: y; *n* = 8, m; *n* = 7, MSO: y; *n* = 12, m; *n* = 11, DNLL: y; *n* = 10, m; *n* = 10. Markers and color-coding as in Figure 2. Colors in **(B,D**, and **E)** represent different stimulation frequencies. Data are shown as mean ± SEM. *p < 0.05, ***p < 0.001.

**Figure 6:**
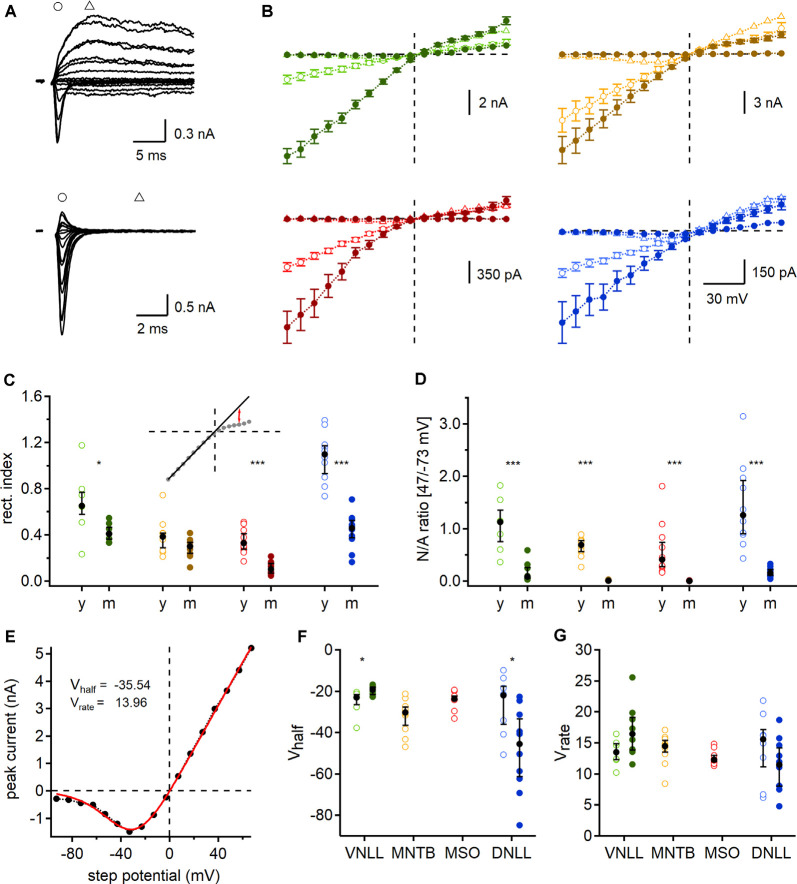
Developmental regulation of AMPAR and *N*-methyl-D-aspartic acid receptor (NMDAR) mediated currents. **(A)** Exemplary currents of a single fiber stimulated P9 (top) and P84 (bottom) MSO neuron at different step potentials. Early, rapid deflection (circles) was considered as a peak of AMPAR mediated EPSC and current 5 ms afterward (triangles) as NMDAR peak. **(B)** AMPAR (circles) and NMDAR (triangles) peak currents of P9/10 (open symbols) and mature (filled symbols) neurons. Data points as mean ± SEM. **(C)** Rectification index (RI) of AMPAR mediated IV-curves. Inset: regression line (black line), fitted at the first eight negative potential values, used to estimate the RI at 47 mV (red arrows). **(D)** Ratio of NMDAR (at 47 mV) to AMPAR (at −73 mV) peak currents (N/A). **(E)** Mean NMDAR IV relationship of P9/10 MNTB neurons fitted with Boltzmann function (red line). *V*_half_ and *V*_rate_ describe the non-linear NMDAR kinetics. **(F,G)** NMDAR voltage-dependent activation in each nucleus and age group given by *V*_half_ (left) and *V*_rate_ (right). Cell numbers per nucleus and age group as in Figure 4. Markers and color-coding as in Figure 2. Black symbols of **C,D,F**, and **G** show median with first and third quartiles. *p < 0.05, ***p < 0.001.

**Figure 7:**
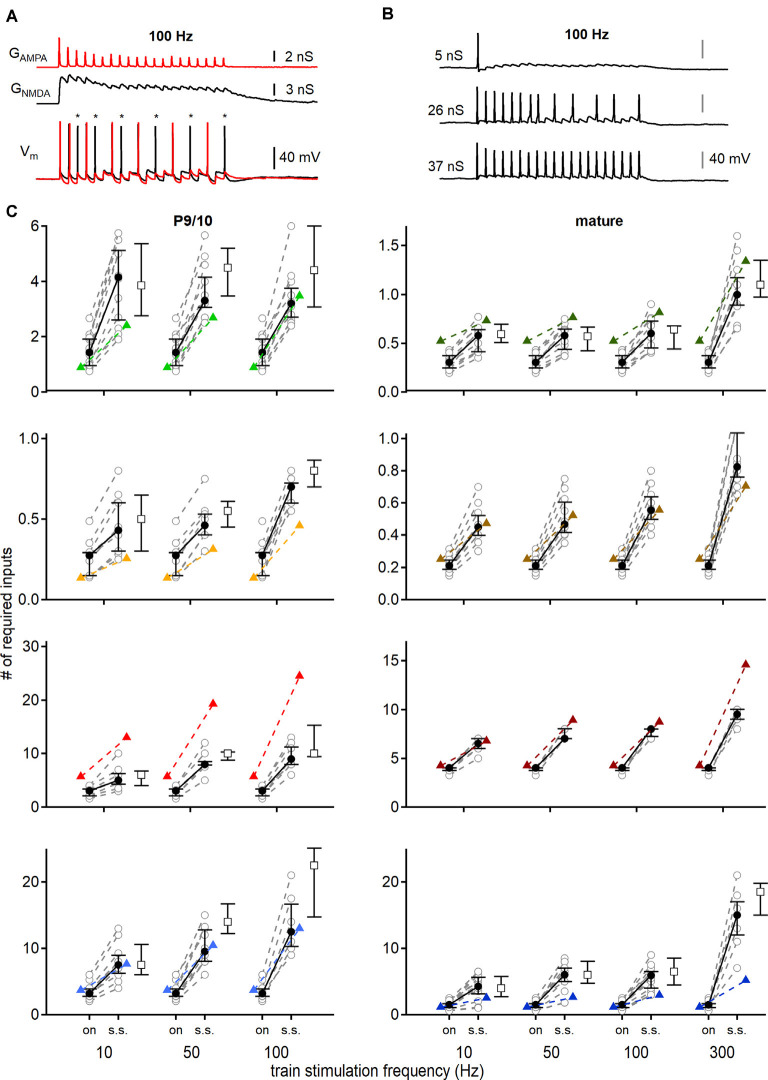
Conductance based input-output functions to estimate the minimal input requirement for action potential generation. **(A)** Stimulation with AMPAR (red) and NMDAR (black) conductance templates (top) and recorded voltage responses (bottom). The addition of NMDAR conductance generated more action potentials (black trace). Red voltage response was evoked by only AMPA EPSGs. Asterisks show additional action potentials when both conductances (AMPAR and NMDAR) were applied. **(B)** Voltage responses of a P69 DNLL neuron to 100 Hz EPSG trains. Scaling the template amplitude to extract the conductance threshold for single action potential generation (top) until all pulses within the train stimulation produced action potentials (bottom). nS values refer to the peak conductance of the first pulse. **(C)** Number of minimally required inputs of P9/10 (left) and mature (right) VNLL, MNTB, MSO, and DNLL neurons for single onset (left symbols, on) and continuous steady-state (right symbols, s.s.) action potential generation, according to firing frequency (10, 50 and 100 Hz, and 300 Hz for mature neurons). Predictions of Figure 5 displayed as colored triangles. Open squares show input requirements by removing the NMDAR conductance. Number of recorded neurons for VNLL: y; *n* = 12, m; *n* = 11, MNTB: y; *n* = 9, m; *n* = 10, MSO: y; *n* = 8, m; *n* = 7, DNLL: y; *n* = 10, m; *n* = 13. Data are shown as median and first and third quartiles. Nuclei color-coded as in Figure 2.

**Figure 8:**
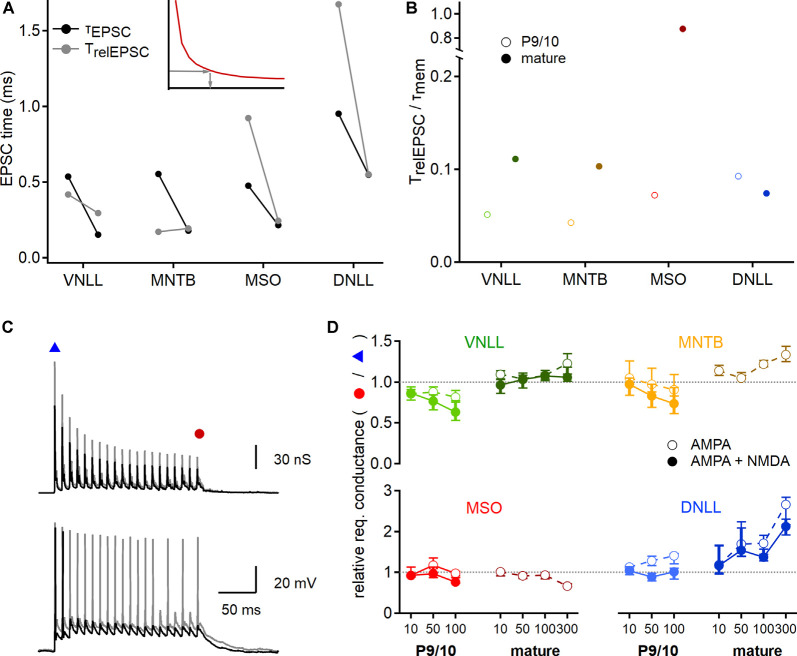
Developmental maturation of postsynaptic integration. **(A)** EPSC time that is integrated to generate an action potential at the conductance threshold. Inset: The relevant EPSC time (*T*_relEPSC_; gray arrows) was estimated from strength-duration curves (red curve), by using the conductance leading to an onset action potential (*y*-axis) to extract the required stimulation time (x-axis). Black symbols show median values of P9/10 (left) and mature (right) fast decay values directly measured from EPSCs (*τ*_EPSC_), used to predict input numbers in Figures 4, 7. Gray symbols point to the relevant EPSC time (*T*_relEPSC_) required to elicit onset action potentials at the conductance threshold, based on recordings shown in Figure 7. **(B)** Ratio of *T*_relEPSC_ to *τ*_mem_ of young (open symbols) and mature (filled symbols) neurons for all nuclei tested. **(C)** EPSG templates (top) were increased to probe conductance thresholds of voltage responses (bottom) at the beginning and the end of the stimulation train. Smaller EPSG generated a single onset action potential (black trace, blue triangle), larger EPSG lead to nearly ongoing action potential generation (gray trace, red circle). **(D)** Ratio of required EPSG peak conductance of last stimulation pulse to onset stimulation pulse for all nuclei and age groups (P9/10: left, mature: right) and at different stimulation frequencies (10, 50, 100 Hz, and 300 Hz for mature neurons). Responses based on AMPAR and NMDAR EPSGs are shown in filled symbols, for AMPAR EPSGs in open symbols. A value above 1 (dotted lines) indicates an increased conductance requirement for steady-state action potential generation. Data are shown as median and first and third quartiles. Cell numbers per nucleus and age group as in Figure 7. Nuclei color-coded as in Figure 2.

## Results

The biophysical activity pattern of P9/10 and mature neurons in the ventral nucleus of the lateral lemniscus (VNLL), medial nucleus of the trapezoid body (MNTB), medial superior olive (MSO) and dorsal nucleus of the lateral lemniscus (DNLL) are established from long step current injections (Banks and Smith, 1992; Forsythe, 1994; Ahuja and Wu, 2000; Scott et al., 2005; Chirila et al., 2007; Porres et al., 2011; Ammer et al., 2012; Franzen et al., 2015; Baumann and Koch, 2017). Our comparative data corroborated the described onset action potentials in MNTB, MSO, and VNLL neurons and the continuous firing from DNLL neurons (Figure 1).

### Sub- and Supra-Threshold Membrane Properties

To compare the membrane properties close to rest in different auditory nuclei and age groups, a small (5 −50 pA dependent on cell type), 250 ms long square pulse was applied 50 times and the response average was used to extract the membrane time constant (*τ*_mem_) and input resistance (*R*_in_; Figure 2A). In all nuclei the *τ*_mem_ accelerated during late postnatal development (VNLL: *p* = 0.00039; MNTB: *p* = 0.019; MSO: *p* = 0.00002; DNLL: *p* = 0.0001, Mann–Whitney *U*). For mature neurons, the fastest *τ*_mem_ was observed in the MSO (0.236 ms) and the slowest in the DNLL (7.423 ms), while VNLL and MNTB neurons displayed an intermediate *τ*_mem_ (2.658 and 2.234 ms, respectively, Figure 2B, top). After developmental maturation, *R*_in_ dropped significantly in all nuclei except the DNLL, corroborating earlier findings (Ahuja and Wu, 2000; Ammer et al., 2012) of an insignificant change in DNLL neurons’ input resistance between P9/10 and P53 (VNLL: *p* = 0.00092; MNTB: *p* = 0.000078; MSO: *p* = 0.000022; DNLL: *p* = 0.156, Mann–Whitney *U*, Figure 2B, middle). Thus, the developmental regulation of *R*_in_ in DNLL neurons might follow other mechanisms compared to the other nuclei. Mature MNTB neurons appeared slightly, but not significantly leakier compared to VNLL neurons (*p* = 0.16, Mann–Whitney *U*), while MSO neurons had the lowest input resistance (3.76 MΩ; Figure 2B, middle). The resting potential of P9/10 neurons was not significantly different between the nuclei (*p* = 0.32, Kruskal–Wallis). Neurons in the VNLL, MNTB, and MSO showed no developmental changes in resting potential (*p* > 0.05, Mann–Whitney *U*). However, mature DNLL neurons rested at more positive values compared to P9/10 neurons (*E*_rest_ = −67.45 mV, *p* = 0.0003, Mann–Whitney *U*, Figure 2B, bottom).

To gain insights into the excitability profile of auditory brainstem neurons, we recorded strength-duration relationships (Figure 3A). The current injection was increased in strength (in steps of 30–100 pA) for 11 different stimulation lengths ranging from 0.1 to 150 ms depending on the cell type. The first current which elicited an action potential was defined as the current threshold for the given pulse duration (Figure 3A, right). This current threshold depended strongly on the stimulation time and showed nucleus and age-dependent adaptations (Figure 3B).

To quantify the strength-duration profiles, we extracted rheobase and chronaxie values (Figure 3C). Rheobase increased during development in MSO (P9/10: 0.49 nA; P73: 4.78 nA, *p* = 0.000012, Mann–Whitney *U*) and MNTB (P9/10: 0.29 nA; P62: 0.53 nA, *p* = 0.016, Mann–Whitney *U*), but not in VNLL and DNLL neurons. Chronaxie was significantly decreased in all nuclei after postnatal development (VNLL: *p* = 0.0000097; MNTB: *p* = 0.000031; MSO: *p* = 0.000012; DNLL: *p* = 0.0086, Mann–Whitney *U*). The acceleration of chronaxie follows from its strong dependency on the *τ*_mem_ (Figure 3D), indicating that the integration time window was largely influenced by the membrane time constant close to the resting state. Since the voltage threshold is another factor that contributes to excitability, we investigated whether it changed during late postnatal development. Indeed, the somatic voltage threshold decreased in mature VNLL (*p* = 0.021, Mann–Whitney *U*) and MSO neurons (*p* = 0.025, Mann–Whitney *U*). In mature neurons, the voltage threshold was comparable between the different nuclei (*p* = 0.13, Kruskal–Wallis, Figure 3E). Notably, the difference between resting potential and voltage threshold decreased in all mature neurons (VNLL: *p* = 0.024; MNTB: *p* = 0.014; MSO: *p* = 0.0008; DNLL: *p* = 0.00004, Mann–Whitney *U*, Figure 3F). Thus, auditory brainstem neurons appear to become intrinsically more excitable.

### Predicting Required Input Fiber Numbers From Excitatory Single Fiber Inputs

Next, we aimed to predict the number of afferent inputs that are required for supra-threshold excitation in the different neuronal populations. Towards this aim, we first recorded pharmacologically isolated EPSCs evoked by minimal fiber stimulation for each nucleus and age group at a holding potential of −93 mV (Figure 4A). Corroborating earlier reports, the EPSC amplitude showed a developmental difference in the VNLL [P9/10: 2.5 nA; P23: 7.73 nA, *p* = 0.00031, Mann–Whitney *U* (Baumann and Koch, 2017)] and the MSO [P9/10: 0.7 nA; P73: 1.88 nA, *p* = 0.00079, Mann–Whitney *U*; Figure 4B (Franzen et al., 2020)]. In the MNTB and DNLL the EPSC size increased slightly, but not significantly (MNTB: P9/10: 9.55 nA; P62: 11.68 nA, *p* = 0.18, DNLL: P9/10: 0.36 nA; P53: 0.55 nA, *p* = 0.063, Mann–Whitney *U*). The EPSC decay time (*τ*_EPSC_) was extracted with a bi-exponential function. The fast decay component (*τ*_fast_; Figure 4C, left data points) and the weighted decay time of the EPSCs (*τ*_weighted_; Figure 4C, right data points) significantly accelerated age dependently in all nuclei except for the fast component in the DNLL. Notably, both *τ*_fast_ and *τ*_weighted_ of mature VNLL, MNTB, and MSO neurons were comparable (*τ*_fast_; *p* = 0.11, *τ*_weighted_: *p* = 0.096, Kruskal–Wallis), while DNLL EPSCs were significantly slower (Figure 4C).

Our next goal was the numerical estimation of minimum afferent fibers that generate temporally precise action potentials. To that end, we compared the EPSC time course with the strength-duration relationship. The strength-duration curves (Figure 3B) were used as a dose-response curve and the median EPSC decay time constant (Figure 4C) as a proxy for stimulation duration. As exemplified in Figure 4D for VNLL neurons, the median of the fast EPSC component was used as a stimulus duration for each individual strength-duration curve to extract its inferred current threshold. Those current thresholds were then compared with the median EPSC amplitude of a single fiber to infer the required input numbers (Figure 4D). Figure 4E displays the estimated input numbers to elicit a single action potential: in P9/10 VNLL one input ensured postsynaptic action potential generation in about 50%, while in mature neurons one input was always sufficient (P9/10: 0.86 inputs; P23: 0.52 inputs, *p* = 0.00013, Mann–Whitney *U*). For MNTB neurons, we predicted larger input portions in mature compared to young animals (P9/10: 0.12 inputs; P62: 0.2 inputs, *p* = 0.00031, Mann–Whitney *U*), although in both groups a single fiber guaranteed postsynaptic firing under resting conditions. For the mature MSO, the increase in EPSC size appeared as one factor to compensate for the enhanced leakiness, as mature neurons required fewer inputs (P9/10: 4.62 inputs; P73: 3.27 inputs, *p* = 0.022, Mann–Whitney *U*). Three times fewer inputs were estimated for mature compared to P9/10 DNLL neurons (P9/10: 3.67 inputs; P53: 1.13 inputs, *p* = 0.000044, Mann–Whitney *U*).

So far, our estimate only applies to the onset of the systems in resting-state. The number of inputs required to excite a neuron supra-threshold during the ongoing activity will depend on the STP of the synaptic inputs. Therefore, we investigated the STP of neurons in the VNLL, MNTB, MSO, and DNLL in P9/10 and mature age groups. To elicit STP, single fiber inputs were stimulated with a 20-pulse train at frequencies of 10, 50, 100, and 300 Hz (Figure 5A). In P9/10 animals, 300 Hz trains could not reliably generate action potentials throughout the stimulus and were omitted from the analysis. The responses to the stimulation train were normalized to the initial EPSC amplitude (Figure 5B) and the steady-state depression was averaged over the last three pulses. The normalized steady-state depression changed significantly in the VNLL and MSO after maturation for all stimulation frequencies (Figure 5C, top). In the MNTB, age-dependent differences in steady-state depression were apparent at high stimulation frequencies. No significant alteration in STP was detected in the DNLL. Inputs to VNLL neurons appear to depress the least, compared to the other matured nuclei. Next, we quantified the decay into depression from the first pulse to steady-state (Figure 5C, bottom). In the VNLL, age did not significantly contribute to decay kinetics, except in high-frequency stimulations (100 Hz, *p* = 0.034, Student’s *t-test*). Moreover, stimulation frequency did not seem to affect the STP decay rate in P9/10 animals. In mature animals, the STP decay was frequency-dependent only in VNLL and MSO neurons (VNLL, P27: *p* = 0.0015; MSO, P80: *p* = 0.0051, Welch’s ANOVA). Taken together, the presence of STP will alter the estimated input requirement for supra-threshold excitation from onset to ongoing activity.

Taking into account the depression of the EPSC size during stimulation trains (Figure 5D, top), we readjusted the minimal requirement of inputs that are predicted to evoke temporally precise action potentials for 20 pulses according to stimulation frequency (Figure 5D, bottom). Figure 5E shows the minimum input requirements for onset and steady-state according to the STD for all four nuclei. In the P9/10 VNLL, multiple input fibers were required for action potential generation, but only one in mature neurons, for up to 100 Hz stimulation frequency. Above 100 Hz, we predicted 1.3 required inputs in mature VNLL neurons. For MNTB neurons, our prediction showed constantly supra-threshold excitation by a single afferent fiber (Figure 5E). In MSO neurons the predicted input requirements became smaller with age but increased with higher stimulation frequency (P9/10 vs. P80; 50 Hz: 21.7–9.5; 100 Hz: 26.6–10.1 inputs, Figure 5E). At mature MSO neurons, 18.1 simultaneous inputs were predicted to generate faithful output at 300 Hz stimulation. In DNLL, the number of required inputs for ongoing output generation significantly decreased (Figure 5E). Mature DNLL neurons appeared easily excited as 2.5–3 inputs for low and 5.1 inputs for high stimulation frequencies were required for temporally precise ongoing firing. Taken together, based on AMPAR mediated currents, steady-state depression increased the numbers of required afferent inputs for faithful action potential generation and late postnatal development renders auditory neurons supra-threshold responsive to a lower number of input fibers.

So far, our estimated number of required input fibers for ongoing action potential generation was based on AMPA mediated currents. However, electrogenic NMDAR components have been described in several auditory brainstem nuclei even in mature stages (Fu et al., 1997; Sivaramakrishnan and Oliver, 2006; Pliss et al., 2009; Ammer et al., 2012; Couchman et al., 2012; Siveke et al., 2018; Winters and Golding, 2018). In some cases, NMDAR mediated currents may have the ability to activate at low potentials and thereby amplify output generation, possibly influencing the number of required fibers for action potential generation during ongoing stimulations. To determine this possible contribution of synaptic NMDAR mediated currents and quantify their voltage dependence we recorded synaptically evoked, single fiber current-voltage relationships in the VNLL, MNTB, MSO, and DNLL for both age groups. The AMPAR mediated component was defined as the rapid current occurring at the onset of stimulation at negative holding potentials and the NMDAR mediated component as the residual current after 5 ms (Figure 6A).

In most cases, the current-voltage relationship showed inward rectification at positive step potentials (Figure 6B). This rectification is an indicator of the AMPAR subunit composition and suggests at least a partial absence of GluR2 (Hollmann et al., 1991; Bowie and Mayer, 1995; Kamboj et al., 1995; Koh et al., 1995). To quantify this rectification and to gain insights into probable developmental changes, we determined the RI, by fitting a regression line to the first eight negative step potentials and calculating the ratio of the measured to the projected values at 47 mV. We report a significant reduction of the RI in all mature neurons except the MNTB (VNLL: *p* = 0.029; MNTB: *p* = 0.95; MSO: *p* = 0.000053; DNLL: *p* = 0.000011, Mann–Whitney *U*). These data corroborated recent reports that MSO neurons undergo loss of GluR2 subunits during late postnatal development (Franzen et al., 2020). Moreover, a developmental change in AMPAR subunit composition can also be expected in the VNLL and DNLL. As no significant change in RI was detected in MNTB neurons, a distinct developmental regulation of AMPAR subunit appears present in the different nuclei in the superior olivary complex.

In all nuclei of P9/10 animals, an NMDAR component was present. This NMDAR component was strongly downregulated in VNLL and DNLL and even absent in mature MNTB and MSO neurons (Figure 6B). To quantify the selective downregulation of the NMDAR component during development, we used the NMDAR and AMPAR current ratio recorded at 47 and −73 mV, respectively (Figure 6D). We detected a significant decrease of NMDA/AMPA ratio in all mature neurons with an apparent absence of traceable NMDAR mediated currents in MNTB and MSO (VNLL: *p* = 0.00062; MNTB: *p* = 0.000022; MSO: *p* = 0.000026; DNLL: *p* = 0.0000011, Mann–Whitney *U*). Interestingly, the NMDA/AMPA ratio was comparable between the two lemniscal nuclei (*p* = 0.51, Mann–Whitney *U*) and between the two nuclei in the superior olivary complex (*p* = 0.167, Mann–Whitney *U*).

Finally, we fitted the non-linear NMDAR mediated IV relationships shown in Figure 6B by Boltzmann functions (Figure 6E) and determined *V*_half_ and *V*_rate_ (Figures 6F,G). Since no NMDAR mediated currents were detected in mature MNTB and MSO neurons, no values were obtained. Minor developmental changes were found for *V*_half_ in the DNLL and VNLL (Figure 6F). Moreover NMDA voltage-dependence in all P9/10 neurons was similar (*V*_half_: *p* = 0.19; *V*_rate_: *p* = 0.28, Kruskal–Wallis), but significantly differed between mature DNLL and VNLL neurons (*V*_half_: *p* = 0.000091; *V*_rate_: *p* = 0.021, Mann–Whitney *U*). In summary, during late postnatal development, synaptic NMDAR and AMPAR mediated currents refine by losing or editing synaptic current components. Also, the presence of NMDAR mediated currents might alter the prediction of the required input number to sustain ongoing action potential generation in response to continuous stimulation.

### Decomposing Influences of EPSC Components on Ongoing Action Potential Generation

To specify the influence of NMDAR mediated currents on the required number of inputs for ongoing action potential generation and to verify the predictions made so far, we used dynamic clamp recordings to simulate synaptic inputs with and without NMDAR component at different conductance levels and stimulation frequencies.

To generate the appropriate conductance templates, we first recorded synaptically evoked AMPAR and NMDAR mediated currents to train stimulations of 10, 50, and 100 Hz for P9/10, and additionally 300 Hz for adult animals, which were isolated from the same cells for every nucleus and age group, and were converted to conductance templates. The peak conductance of every pulse was adjusted to the average, normalized STP levels, as shown in Figure 5B. For the linear and non-linear voltage dependence of synaptic currents, we used the data presented in Figure 6.

Stimulations were performed consecutively with an injection of AMPA EPSGs with or without NMDA conductances (Figure 7A, top). Cell responses were recorded and synaptic contributions to action potential numbers were extracted (Figure 7A, bottom; asterisks). Additionally, the conductance threshold for single and continuous action potential generation was measured, by up- and downscaling template amplitudes (Figure 7B). Thus, we defined minimum input requirements for temporally precise action potential generation at different parts of the train stimulation with different sets of synaptic conductances (Figure 7C).

In P9/10 neurons the dynamic clamp data verified that more inputs were required for ongoing action potential generation compared to the onset; except for MNTB neurons where one input was sufficient to sustain 100 Hz stimulation frequencies at onset and during 20 pulses (100 Hz; VNLL: *p* = 0.0000028; MNTB: *p* = 0.1; MSO: *p* = 0.00067; DNLL: *p* = 0.00011, Mann–Whitney *U*; Figure 7C, left). For all P9/10 neurons the synaptic NMDAR component amplifies action potential generation at stimulation frequency above 50 Hz (VNLL: 50 Hz, *p* = 0.00098; 100 Hz, *p* = 0.002, MNTB: 50 Hz, *p* = 0.0078; 100 Hz, *p* = 0.016, MSO: 50 Hz, *p* = 0.0078; 100 Hz, *p* = 0.0078, DNLL: 50 Hz, *p* = 0.002; 100 Hz, *p* = 0.043, Wilcoxon signed-rank test; Figure 7C, left).

In mature VNLL neurons at high frequencies, one input was barely able to sustain faithful transmission, as 5 out of 11 neurons required more than the average AMPAR and NMDAR EPSGs to generate action potentials (Figure 7C, right). The NMDA component appeared to be of relevance in the VNLL since 7 out of 11 neurons required more than a single input to fire only with AMPAR EPSGs (300 Hz, *p* = 0.0039, Wilcoxon signed-rank test). Comparable in MNTB 3 out of 10 neurons could not sustain faithful transmission at 300 Hz (Figure 7C, right). That a small number of MNTB neurons failed faithful one-to-one transmission is in agreement with *in vivo* recordings (Guinan and Li, 1990; Kopp-Scheinpflug et al., 2003; Lorteije et al., 2009). As, we did not detect synaptic NMDAR mediated currents in MNTB neurons of this age group, no additional synaptic amplification was present. For the mature MSO, we corroborated the findings of Couchman et al. (2010) that only a low number of input fibers generate a faithful action potential from rest. However, we report that more than nine inputs were required for faithful, high-frequency supra-threshold information transfer (Figure 7C, right). Again, as no synaptic NMDAR current was detected in adult MSO neurons, these currents could not supply additional amplification for action potential generation in this system. In agreement with *in vivo* (Kelly and Kidd, 2000; Siveke et al., 2018, 2019) and *in vitro* data (Porres et al., 2011; Ammer et al., 2012) our dynamic clamp results verified that the synaptic NMDAR component contributes to action potential generation in mature DNLL (50 Hz, *p* = 0.031; 100 Hz, *p* = 0.031; 300 Hz, *p* = 0.044, Wilcoxon signed-rank test; Figure 7C, right). In the mature DNLL, our data predicted that a high number of inputs (*n* = 15) was needed for faithful and temporally precise supra-threshold synaptic information transfer at rates of 300 Hz (Figure 7C, right). Even more so as about half of neurons (6/13) could not spike at every pulse of the stimulation train, because they underwent a depolarization block.

This study aimed to predict input requirements for auditory brainstem neurons by comparing the size of single fiber EPSCs with current thresholds described in the strength-duration curves of Figure 3B. Comparing the initial predictions of onset firing with those recorded under dynamic clamp conditions, only DNLL and mature MNTB neurons were described accurately (DNLL; P9/10: *p* = 0.62; P56: *p* = 0.83, MNTB; P54: *p* = 0.39, Mann–Whitney *U*; Figure 7C).

### Efficacy of EPSC Integration and Postsynaptic Contributions

Next, we seek to understand the source of the discrepancy between the approximated number of inputs for action potential generation estimated from the dynamic clamp recordings and the predictions based on EPSC decay time and strength-duration relationships (Figures 4, 5). A critical value for the initial estimates (Figures 4, 5) was the use of the EPSC decay time constant as a proxy for stimulation time. To validate this assumption, we now inferred the relevant time of the EPSC for action potential generation and compared them to the EPSC decay time constant. Towards this end, the dynamic clamp threshold of EPSGs was converted into a current threshold taking the synaptic reversal potentials into account. This dynamic clamp derived current threshold was used in conjunction with the strength-duration relationships to extract the relevant EPSC time (*T*_relEPSC_; Figure 8A inset). Thus, we estimated the relevant EPSC time for action potential generation. In P9/10 VNLL and MNTB, the *τ*_EPSC_ was larger and in MSO and DNLL smaller than the *T*_relEPSC_ (Figure 8A). In mature stages, only VNLL neurons required a longer *T*_relEPSC_ compared to *τ*_EPSC_ to drive action potential generation. Taken together, the relative fraction of integrated EPSC time changed during late postnatal development as in mature MNTB, MSO, and DNLL neurons the *τ*_EPSC_ matches well with *T*_relEPSC_.

An important aspect of synaptic integration is the relation between the EPSC time course and the membrane time constant (*τ*_mem_). At the beginning of a current step, a large part of the current flow is used to charge the cell membrane while at the end of the step the current flows through the resistive membrane element. Thus, at small *T*_relEPSC_/*τ*_mem_ ratios most current is used to charge the cell membrane and at large ratios, most of the current is integrated at the steady-state through the resistive element. Calculating the *T*_relEPSC_/*τ*_mem_ ratio showed that only in mature MSO neurons the EPSC time course matches closely the *τ*_mem_ and therefore is mostly integrated *via* the resistive element (Figure 8B). All other ratios were <0.15. Especially low values of the *T*_relEPSC_/*τ*_mem_ ratio were found in young neurons of VNLL (0.05) and MNTB (0.04; Figure 8B). Thus, in these systems, most of the synaptic current that leads to action potential generation is used for charging the neuron. In VNLL, MNTB, and MSO the adult values of the *T*_relEPSC_/*τ*_mem_ ratio were larger than the young ones, indicating that integration properties increase during late postnatal development in the auditory brainstem neurons. In DNLL the decay ratio appeared similar in young and mature stages.

The predictions based on EPSC decay time constant and dynamic clamp recordings were different between the onset and the ongoing action potential generation (Figure 7C). Therefore, the postsynaptic neuron might change the efficacy of action potential generation during ongoing activity. To quantify these postsynaptic adaptations, we extracted the conductance threshold that was required for the first and the last simulated EPSG in the stimulation train (Figure 8C). A ratio below one indicated that the last EPSG threshold in the train is smaller than for the onset EPSG threshold, indicative of a facilitator postsynaptic effect. Indeed, the fraction of the required conductance between onset and ongoing supra-threshold excitation appeared to be age, nucleus, and frequency-dependent but was largely unaffected by the presence of NMDAR conductances (Figures 8C,D). In P9/10 VNLL neurons, action potential generation was facilitated during high-frequency input trains, while in mature cells no frequency dependence was observed. Young MNTB neurons behaved similarly as P9/10 VNLL neurons with a small facilitating effect. Neurons in the mature MNTB, however, showed a suppressive effect of ongoing activity to action potential generation. This suppressive effect likely explains the difference between AMPAR current based estimates and dynamic clamp (Figure 7C). High input frequencies facilitated action potential generation in MSO neurons, a finding in agreement with the resonance properties of these cells (Fischer et al., 2018). Strong frequency-dependent suppression of action potential generation was evident in the mature DNLL. In summary, the postsynaptic neurons differentially modulate the efficacy of EPSGs in action potential generation during ongoing activity.

## Discussion

Here, we use the strength-duration relationship of current injections to quantify action potential generation and measure synaptic transmission to illuminate the synaptically evoked input-output functions of four different auditory brainstem nuclei in two age groups. Combining both measurements, we infer the minimal number of synchronously active input fibers for temporally precise action potential generation. Besides estimating the required input numbers, the strength-duration relationship served as a dose-response curve to infer the relevant EPSC times. We find that auditory brainstem neurons integrate different fractions of the EPSC for supra-threshold activity. We infer that during development, fewer input fibers are required to drive action potentials, even though the input resistance and the electrogenic NMDAR component are reduced. To sustain ongoing action potential generation during stimulation trains the number of required input fibers must increase to compensate for synaptic depression. Dynamic clamp recordings corroborated the impact of different synaptic current contributions on minimally required input numbers and allowed the estimation of the relevant EPSC time for action potential generation. Thereby, we also observed postsynaptic contributions increasing and decreasing the required number of input fibers for action potential generation during stimulation trains.

### Age-Related Differences

Neurons in the auditory brainstem are well-known to undergo refinements during late postnatal development. These refinements include biophysical, synaptic, and morphological properties. However, our comparative approach shows that not all nuclei regulate physiological parameters in the same manner. For example, we corroborate the finding (Ahuja and Wu, 2000; Ammer et al., 2012) that input resistance in DNLL neurons remains stable from before hearing onset to maturity. However, only in the DNLL, the resting potential becomes significantly less negative during late postnatal development, while the difference between resting potential and action potential voltage-threshold becomes less in all nuclei tested. Thus, the development process seems to render auditory brainstem neurons intrinsically more excitable. An increase in synaptic EPSC size is restricted to the VNLL and MSO, while MNTB input size remained largely unchanged, in line with earlier reports from rats (Taschenberger and von Gersdorff, 2000) but contrary to mice (Youssoufian et al., 2005). Overall, we describe differential developmental regulations in distinct auditory brainstem nuclei. We suggest furthermore, that these differential regulations arise from different regulatory cues driving development, such as genetic programs, activity (Clause et al., 2014; Franzen et al., 2020), and acoustic experience (Kapfer et al., 2002).

A common postnatal change was the acceleration of the membrane time constant at resting potentials. This reduction is likely based on the decrease in input resistance (Magnusson et al., 2005; Scott et al., 2005; Chirila et al., 2007; Franzen et al., 2015) or a reduction in cell size (Franzen et al., 2015). In conjunction with the acceleration of the membrane time constant, the chronaxie decreased overall. These accelerations indicate a significant reduction in the integration time window for auditory brainstem nuclei in general. Thus, irrespective of additional changes and regulatory cues the speed of postsynaptic integration accelerates and shortens the integration time windows. The acceleration of the membrane time constant was thereby larger than changes in EPSC kinetics. In respect to the EPSC time course, a faster membrane time constant will permit less EPSC time to charge the cell membrane and more to be integrated *via* the input resistance. This change might lead to a larger integration of excitatory with inhibitory or other modulatory inputs in steady-state of the membrane biophysics.

Parallel to the common reduction in membrane time constant, a general reduction of the required input fibers for action potential generation was observed. This drop appears counterintuitive as most neurons become leakier and the excitatory NMDAR component is downregulated. However, several other factors compensate for this apparent reduction in excitability. First, the difference between resting potential and action potential voltage threshold becomes smaller in all nuclei. Second, the EPSC size can increase. Third, the acceleration of the membrane time constants can lead to a larger integrated fraction of the relevant EPSC time. Together these alterations allow increasing the efficacy of the EPSC towards the generation of supra-threshold events.

An increase in efficacy and a reduction in the number of required synchronous inputs to drive action potential generation may be one additional mechanism to compensate for the developmental loss of inputs during the pruning period. During postnatal development, a pruning period removes inputs from dendrites to sharpen the tonotopical map (Kim and Kandler, 2003). While for MNTB neurons with one-to-one connection this will be less important, other nuclei might refine tonotopically in such a manner. By losing input fibers these nuclei require therefore compensatory alterations for maintaining action potential generation. In addition to the loss of excitatory inputs, developmental strengthening of inhibition by re-localization synapses (Kapfer et al., 2002), becoming hyperpolarizing (Ehrlich et al., 1999; Blaesse et al., 2006) and stronger (Kim and Kandler, 2003; Kotak and Sanes, 2014; Winters and Golding, 2018) needs to be compensated. To overcome the enhanced inhibition a developmental increase in excitability exerted by a combination of the described cellular features might be necessary.

### Nuclei Specificities and Structure-Function Consideration

It is tempting to compare the VNLL and the MNTB due to their similar structure-function relationship. Both receive large excitatory somatic synapses (Held, 1893; Stotler, 1953; Forsythe, 1994; Adams, 1997; Berger et al., 2014) and generate rapid, temporally precise feed-forward inhibition. Despite the similarity of the systems, we found discrepancies in postnatal development indicating differences in the processing of input-output functions. MNTB neurons increase the rheobase while VNLL neurons increase the EPSC size, decrease the RI, retain a small NMDAR current and the STD is weak below 300 Hz stimulation frequencies. Thus, in both functionally similar systems, late postnatal development regulated distinct parameters. However, postsynaptic adaptive mechanisms during train stimulations appear similar, as for young VNLL and MNTB a supportive and in adult neurons a slight suppressive effect of high-frequency stimulation on action potential generation was observed. Overall, both systems are well developed in mature animals to sustain a one-to-one input-output function for stimulation frequencies up to 300 Hz. Thereby a smaller depression and the smaller postsynaptic adaptive changes during ongoing activity in VNLL neurons might counterbalance the initial smaller amplitude. Nevertheless, at 300 Hz about 50% of the VNLL neurons will not follow the simulation faithfully, while in the MNTB this ratio drops to 30%. Thus, a single MNTB input outperforms a single VNLL input, especially during ongoing, synaptic high-frequency stimulation. This difference might be systemically relevant. VNLL neurons phase lock to the sound onset and follow modulation frequencies of 10–300 Hz ideal for coding envelope sound structures (Zhang and Kelly, 2006; Recio-Spinoso and Joris, 2014) to generate rapid onset inhibition in the inferior colliculus (Pollak et al., 2011; Moore and Trussell, 2017). The more transient responsiveness of VNLL neurons may be helpful to cost-effectively relay their octopus cell inputs, known to transmit broadband sound onsets (Smith et al., 2005; Recio-Spinoso and Rhode, 2020), to the inferior colliculus. MNTB neurons phase lock to stimulations of more than 500 Hz with low failure rates (Guinan and Li, 1990; Lorteije et al., 2009). The phase-locked glycinergic inhibition of the MNTB is also involved in processing low-frequency sound sources in the MSO (Brand et al., 2002; Pecka et al., 2008; Goldwyn et al., 2017) based on temporal disparities in gerbils. To maintain sound source localization the MNTB is required to sustain input-output functions at higher frequencies than VNLL neurons. It is therefore hypothesized that the subtle differences between VNLL and MNTB are functionally relevant.

Previously it has been reported that MSO neurons require a small number of inputs to generate action potentials at rest (Couchman et al., 2010). We corroborate and extend this finding with a different experimental approach. As excitatory MSO inputs substantially depress the required number of simultaneously active fibers increases from about four to ten to sustain supra-threshold excitation during ongoing activity. Ten inputs have still to be regarded as a lower estimate. As hyperpolarizing inhibitory inputs (Magnusson et al., 2005) are integrated to provide proper binaural coding (Brand et al., 2002; Pecka et al., 2008; Myoga et al., 2014; Goldwyn et al., 2017), more excitation will be required to reach action potential threshold. Thus, more inputs will have to converge to generate an appropriate safety factor to detect interaural time differences. Our figures so far present a minimal requirement during physiologically relevant excitatory sound stimulation. However, at high stimulation frequencies, the synaptic depression appears to be slightly counterbalanced by postsynaptic adaptive mechanisms enhancing the efficiency of output generation. Membrane resonance in MSO neurons occurs at about 300 Hz (Fischer et al., 2018) and could constitute the underlying membrane phenomena for this counterbalance.

DNLL neurons become highly excitable during late postnatal development. The DNLL requires an unexpectedly low number of inputs for onset action potential generation at rest. In adult animals, this low number is partially explained by the small potential difference between rest and action potential threshold. Some of the recorded DNLL neurons were discarded from further analysis, as they were spontaneously active. Thus, DNLL neurons require very little excitatory drive to be brought above the voltage threshold. Moreover, NMDAR mediated currents support action potential generation (Fu et al., 1997; Kelly and Kidd, 2000; Porres et al., 2011; Ammer et al., 2012; Siveke et al., 2018). Taken together, DNLL neurons rest close to the action potential threshold, and small AMPAR currents that ride on a slowly integrated depolarizing NMDAR current trigger action potential generation at input frequency up to 100 Hz. Possibly this high excitability is required to overcome hyperpolarizing inhibition during sound stimulations (Yang and Pollak, 1994; Ammer et al., 2015; Siveke et al., 2019) or, if present in a tonic way, due to some spontaneously active contralateral DNLL neurons (Bajo et al., 1998). Despite this high excitability and the fact, that some of the DNLL neurons can fire high frequencies *in vivo* (Kuwada et al., 2006; Siveke et al., 2006) and in response to *in vitro* current injections (Ahuja and Wu, 2000; Ammer et al., 2012), their maximal output frequency appears limited by the severe synaptic depression and postsynaptic adaptive mechanisms. The latter may include sodium channel inactivation during high-frequency stimulation trains. These suppressive mechanisms might be required to reduce the risk of hyper-excitability of DNLL neurons. Despite these neurons being regarded as integrating cells, the ratio between *T*_relEPSC_ and *τ*_mem_ indicates that under resting conditions most of the synaptic current driving action potential generation is mainly used to charge the membrane.

In summary, our study shows how distinct auditory nuclei are individually refined during development for specific biophysical and synaptic integration behaviors matching the functional needs within their structure-function relationship.

## Data Availability Statement

The raw data supporting the conclusions to this article are available from the corresponding author upon request.

## Ethics Statement

Ethical review and approval was not required for the animal study, because the german law permits organ extraction from rodents after they have been euthanized. This is regarded as an organ extraction and does not require the approval of an external committee. The university’s animal wellfare agent has approved our procedures and we report the number of animals used within the University.

## Author Contributions

FF: conceptualization. NK and FF: design of experiments, data handling, analysis and interpretation, and writing—original draft. NK, LF, and FF: writing—review and editing. All authors contributed to the article and approved the submitted version.

## Conflict of Interest

The authors declare that the research was conducted in the absence of any commercial or financial relationships that could be construed as a potential conflict of interest.
